# Hematuria Management in Patients on Antiplatelet Medications After Acute Coronary Syndrome: A Review of the Current Evidence and Recommendations

**DOI:** 10.31083/RCM46334

**Published:** 2026-03-06

**Authors:** Ioannis Loufopoulos, Konstantinos Kapriniotis, Barbara Fyntanidou, Aikaterini Apostolopoulou, Athina Nasoufidou, Panagiotis Stachteas, Efstratios Karagiannidis, Efstathios Papaefstathiou

**Affiliations:** ^1^Urology Department, Ipswich Hospital, IP4 5PD Ipswich, UK; ^2^Department of Urology, Whipps Cross Hospital, Barts Health NHS, E11 1NR London, UK; ^3^Department of Emergency Medicine, Aristotle University of Thessaloniki, AHEPA General University Hospital of Thessaloniki, 54636 Thessaloniki, Greece; ^4^2nd Cardiology Department, Ippokrateio General Hospital of Thessaloniki, Aristotle University of Thessaloniki, 54642 Thessaloniki, Greece; ^5^Urology Department, Russell's Hall Hospital DGFT, DY1 2HQ Dudley, UK

**Keywords:** hematuria, bleeding, hemorrhage, hemodynamic, acute coronary syndrome, antiplatelet, anticoagulant

## Abstract

Hematuria is a frequent urological presentation, particularly in patients with significant cardiovascular comorbidities who receive dual antiplatelet therapy (DAPT) after acute coronary syndrome (ACS). Managing hematuria in this high-risk population poses a unique clinical challenge, requiring a careful balance between thrombotic and bleeding risks. This review summarizes current evidence and provides practical recommendations for the multidisciplinary management of hematuria in patients on antiplatelet medications following ACS. Relevant literature and international guideline recommendations from urology, cardiology, and emergency medicine were reviewed, focusing on diagnostic evaluation, hemodynamic assessment, modification of antiplatelet therapy, surgical considerations, and reversal strategies. The management pathway begins with a prompt assessment of hemodynamic stability, hematuria severity, and underlying cause. Conservative measures include catheterization, bladder irrigation, and correction of coagulation disorders. The diagnostic evaluation should not be delayed, as up to 24% of cases of visible hematuria in this population are due to malignancy. Antiplatelet management depends on bleeding severity and thrombotic risk: mild bleeding generally allows continuation of DAPT; moderate bleeding may warrant temporary cessation of aspirin; severe bleeding often requires de-escalation to monotherapy; life-threatening bleeding necessitates immediate discontinuation of all antiplatelets. Interventional options—ranging from endoscopic clot evacuation to selective arterial embolisation—should be tailored to the stability and cardiovascular risk of the patient. Resumption of antiplatelet therapy should occur as early as clinically feasible, ideally within 48 hours, with de-escalated regimens considered for patients with a high bleeding risk. Hematuria in post-ACS patients on antiplatelets requires an individualized, multidisciplinary approach to optimize hemostasis without compromising cardiovascular protection. Early diagnosis of underlying urological pathology is essential, and both bleeding severity and ischemic risk should guide antiplatelet modification therapy. Evidence supports early specialist involvement, adherence to structured risk-adapted protocols, and judicious use of conservative or interventional measures to improve outcomes.

## 1. Introduction 

In both emergency and elective urology practice, the management of complex patients with multiple comorbidities is increasingly frequent. Hematuria is a common presentation, often occurring in individuals with significant cardiovascular diseases who are receiving antithrombotic medication [1]. Meanwhile, it is not uncommon for the urologist to face the dilemma of withholding these medications in the acute setting or in preparation for the definitive surgical management of the underlying cause of bleeding, such as in urothelial tumors or obstructed kidneys. The associated risks of withholding antiplatelet or anticoagulant medications directly relate to the nature and timing of the cardiovascular event, along with other factors such as comorbidities and the suspected cause of hematuria [2]. Among this group of patients, people with recent acute coronary syndrome (ACS) who typically receive dual antiplatelet treatment (DAPT) are the most challenging to manage, both due to the considerable and detrimental thrombotic risk that comes with interrupting DAPT, but also due to the prolonged course of action of these medications, which can make hemostasis challenging for surgical intervention in an otherwise very high-risk population [3].

Most patients presenting with ACS typically undergo invasive or surgical reperfusion treatment (usually percutaneous coronary intervention (PCI) or, less commonly, coronary artery bypass grafting (CABG)) either in the emergency setting or during the index admission. Simultaneously, these patients are initially treated with DAPT, ideally with a potent P2Y purinoceptor 12 (P2Y12) inhibitor such as prasugrel or ticagrelor. The recommended duration of DAPT by both the European Society of Cardiology (ESC) and a collaboration of relevant American bodies (American College of Cardiology (ACC)/American Heart Association (AHA)/American College of Emergency Physicians (ACEP)/National Association of EMS Physicians (NAEMSP)/Society for Cardiovascular Angiography and Interventions (SCAI)) is 12 months, followed by indefinite admission to a single-agent therapy in the majority of cases [4, 5]. Aspirin is the cornerstone of early ACS management, from the pre-hospital setting through secondary prevention after reperfusion. Aspirin exerts the associated antithrombotic effect mainly by inhibiting the synthesis of thromboxane A2 (TXA2) [6]. However, despite the reduced bleeding risk, aspirin monotherapy has a limited role in the acute management of ACS and the first months post-ACS and should always be used as part of DAPT [4, 5].

Different P2Y12 inhibitors have been utilized as part of DAPT and later on as monotherapy for secondary prevention. There is strong evidence supporting the use of DAPT based on the potent prasugrel (particularly in the setting of PCI) and ticagrelor as opposed to clopidogrel for the first 12 months after ACS/PCI. Conversely, potent P2Y12 inhibitors are associated with increased bleeding risk compared to clopidogrel, both as add-on (to aspirin) treatment and as monotherapy [7, 8]. DAPT with a (preferably) potent P2Y12 inhibitor is absolutely critical for the first 30 days following ACS in patients undergoing PCI; however, recent evidence suggests that more conservative strategies, with regard to bleeding risk, such as switching to a less potent P2Y12 inhibitor as part of DAPT or continuing with a potent P2Y12 monotherapy, could be considered after 1–3 months based on a risk/benefit assessment on an individualized basis [9].

Hematuria can range from non-visible or transiently visible blood in urine to heavy bleeding with a significant hemoglobin reduction that requires transfusion and urgent hemostasis. Compared to other sources, such as the gastrointestinal tract, hematuria is a less common cause of catastrophic bleeding with hemodynamic instability. However, hematuria episodes can cause significant morbidity with recurrent hospitalizations in a generally deconditioned group of patients [10]. The severity and prognosis of hematuria, particularly in patients on antithrombotic treatment, largely depend on the underlying cause and range from self-limiting episodes of bleeding in the context of easily reversible causes, such as urinary tract infection, to severe and recurrent bleeding not responding to conservative management in patients with urological cancers, severe benign prostatic enlargement, or vascular malformations [11]. In such circumstances, when the interruption of a regimen or the undertaking of interventional procedures is under consideration, management decisions must be reached through consultation with a multidisciplinary team, including urologists, cardiologists, hematologists, and anesthetists.

Therefore, this review aimed to synthesize the current evidence on the management of this complex patient population and to propose a rational, evidence-based approach to guide urologists and other healthcare professionals involved in the care of these patients.

## 2. Literature Review

A comprehensive literature search was conducted in the PubMed, Scopus, and Web of Science databases to identify relevant studies examining the management of hematuria in patients receiving anticoagulant or antiplatelet therapy. The search included combinations of the following keywords: hematuria, bleeding, acute coronary syndrome, desmopressin, antiplatelet treatment, anticoagulant, tranexamic acid, aspirin, and P2Y12 inhibitors. No language or time restrictions were applied to ensure broad coverage of available evidence. Reference lists of included articles and relevant reviews were also screened to identify additional studies. Both clinical trials and observational studies evaluating management strategies, outcomes, or complications related to antiplatelet therapy and hemostatic agents in the context of ACS were considered eligible for inclusion.

## 3. Rationale for Hematuria Management in ACS Patients

A thorough clinical assessment of both hematuria and hemodynamic stability represents the initial step in the evaluation and management of macroscopic hematuria in patients receiving antiplatelet therapy for ACS. This evaluation is critical in determining the urgency of urological intervention and whether management should be conducted on an inpatient or outpatient basis. This decision will, in turn, influence any potential modifications or temporary discontinuation of antiplatelet therapy, as well as the timing and setting for further diagnostic procedures.

Subsequently, a systematic investigation into the underlying urological causes of hematuria is warranted. Based on the diagnostic findings, a multidisciplinary team—comprising cardiology and urology specialists—can establish an individualized management plan that carefully weighs the risks of cardiovascular events, hemorrhagic complications, and progression of the underlying urological pathology.

Lastly, reaching consensus on the optimal timing for resuming antiplatelet therapy, including agent selection and duration, is essential, based on the risk stratification and clinical status of the patient.

### 3.1 Assessment of Hematuria

#### 3.1.1 Initial Assessment and Hemodynamic Considerations in Hematuria Management

Before evaluating the etiology of hematuria, assessing the hemodynamic stability of patients is imperative. Moreover, hemodynamic monitoring is essential in all patients presenting with frank hematuria, as clinical deterioration may occur suddenly and unpredictably.

In the emergency setting, particularly among outpatients presenting to the Accident and Emergency (A&E) department, hematuria may not always be clinically apparent. This is especially the case in patients without a urinary catheter or in those with altered mental status, such as unconscious or cognitively impaired individuals. As outlined in the European expert consensus on emergency urological care, the initial assessment in the A&E should begin with monitoring the vital signs, a focused clinical history, and a physical examination in accordance with the ABCDE protocol: airway, breathing, circulation, disability (neurological assessment), and exposure [12]. Signs of major bleeding and hemodynamic stability should be primarily assessed, and the massive transfusion protocol should be activated when indicated [13].

In cases where hematuria is already established and no indwelling catheter is present, urethral catheterization is indicated. A three-way Foley catheter should be inserted, and a manual bladder washout should be performed to assess bleeding volume and characteristics and to relieve potential urinary retention caused by clot obstruction. Continuous bladder irrigation (CBI) should be initiated in cases of gross hematuria to prevent clot formation and catheter blockage. Meanwhile, monitoring urine output is also critical for assessing hydration status and renal function, although accurate measurement can be challenging during irrigation. In such cases, urine volume must be calculated by subtracting the instilled irrigation fluid from the total collected output [10].

#### 3.1.2 Initial Assessment in Hematuria

The initial diagnostic workup for patients presenting with hematuria should include a full blood count, renal function tests, coagulation profile, and blood gas analysis. Renal function assessment, including serum urea, creatinine, and electrolytes, is essential, as this assessment not only aids in identifying any underlying causes, such as obstructive uropathy, but also informs subsequent diagnostic and therapeutic strategies. Renal impairment may point toward obstructive pathologies (*e*.*g*., stones, tumors) or chronic kidney disease as contributing factors to hematuria. Moreover, renal impairment has practical implications for the administration and dosing of nephro-excreted medications, including anticoagulants [14]. The coagulation profile should include activated partial thromboplastin time (aPTT), prothrombin time (PT)/international normalized ratio (INR), and fibrinogen levels to identify any underlying coagulopathy that may exacerbate bleeding or complicate intervention [14].

Imaging plays a critical role in the early evaluation. Indeed, bedside ultrasonography is typically the first-line modality in the emergency setting due to its availability and rapid execution, providing immediate information on bladder fullness, hydronephrosis, or gross anatomical abnormalities [12]. In cases of major hemorrhage of unknown etiology, contrast-enhanced computed tomography (CT) of the abdomen and pelvis, or whole-body CT angiography, if indicated, is the gold standard for urgently localizing the bleeding source [12].

In known cases of hematuria, an assessment of post-void residual volume is recommended via ultrasound before catheterization to exclude urinary retention, which may complicate both diagnosis and management [10].

Further evaluation of the underlying urological cause of hematuria can be undertaken in either the inpatient or outpatient setting, depending on the hemodynamic stability and overall clinical status of the patient.

#### 3.1.3 Classification of Hematuria in the ACS Setting

Clinicians are often required to differentiate between frank macroscopic hematuria and milder presentations, such as microscopic hematuria, which is defined by clear diagnostic criteria: the presence of more than 3 red blood cells per high-power field (RBCs/HPF) on urine microscopy [15]. In contrast, macroscopic hematuria refers to visibly discolored urine containing blood. Notably, as little as 1 mL of blood can alter urine color. The presence of bright red urine or visible clots is highly suggestive of bleeding from the lower urinary tract. In contrast, “cola-colored” urine may indicate glomerular pathology [16]. Bright red urine is typically associated with arterial bleeding, whereas darker shades, such as Bordeaux or dark red, are suggestive of venous bleeding. A dark brown or black discoloration often indicates older blood. Other differential diagnoses of hematuria include myoglobinuria or hemoglobinuria, which can present with dark red urine, as well as certain foods and medications that may alter urine color [17].

The International Society on Thrombosis and Hemostasis (ISTH) defines major bleeding as symptomatic hemorrhage in a critical organ or space, a hemoglobin drop of ≥20 g/L, or the need for transfusion of ≥2 units of red blood cells [18]. Additionally, the American College of Cardiology includes hemodynamic instability in its criteria for major bleeding, defined by systolic blood pressure <90 mmHg, a drop of ≥40 mmHg from baseline, or orthostatic hypotension (≥20 mmHg systolic or ≥10 mmHg diastolic drop upon standing) [19].

The Haute Autorité de Santé (HAS) categorizes bleeding severity based on urgency and required interventions. Severe bleeding is characterized by hemodynamic instability (*e*.*g*., systolic Blood Pressure <90 mmHg or Mean Arterial Pressure <65 mmHg), uncontrolled external hemorrhage, the need for urgent procedures (*e*.*g*., interventional radiology or surgery), the need for transfusion, or a life-threatening risk [20, 21].

In the context of gastrointestinal bleeding, several validated risk stratification tools guide management in anticoagulated patients. The Glasgow–Blatchford score includes hemoglobin, blood urea nitrogen, systolic blood pressure, heart rate, and clinical symptoms such as melena and syncope, as well as comorbidities such as liver disease or heart failure [22]. The Rockall score incorporates patient age, presence of shock, endoscopic findings, and underlying comorbidities to predict 30-day mortality [22]. However, no equivalent scoring system currently exists for stratifying hematuria by mortality risk. Thus, existing bleeding scores from cardiology may offer a useful framework for managing hematuria in patients with ACS.

International guidelines categorize bleeding severity into four groups to guide treatment decisions [23, 24]:

Mild bleeding: Requires medical attention but not hospitalization (*e*.*g*., gingival bleeding, epistaxis, hematochezia).

Moderate bleeding: Results in a hemoglobin reduction of >3 g/dL; the patient remains hemodynamically stable but requires hospital admission, as seen in certain Gastrointestinal [GI] bleeding cases.

Severe bleeding: Defined by a hemoglobin decrease of >5 g/dL; hospitalization is required, although the patient remains stable. Frank hematuria usually falls within this category.

Life-threatening bleeding: Associated with hemodynamic instability and an immediate risk to life.

### 3.2 Initial Management Plan

#### 3.2.1 Hemoglobin Target Levels

The ACC guidelines recommend maintaining a target hemoglobin (Hb) level of 10 g/dL in patients with ACS and anemia, whether chronic or acute, provided the patients are not actively bleeding [5]. Both chronic and acute anemia are associated with adverse cardiovascular outcomes, likely due to impaired myocardial oxygen delivery, increased myocardial oxygen demand, and diminished efficacy of antithrombotic therapies or surgical interventions [5]. The Myocardial Ischaemia and Transfusion (MINT) trial evaluated the impact of transfusion thresholds in 3504 patients presenting with ST-elevation myocardial infarction (STEMI) or non-ST-elevation myocardial infarction (NSTEMI) and hemoglobin levels below 10 g/dL. Patients were randomized to either a restrictive transfusion strategy (triggered at Hb <7–8 g/dL) or a liberal strategy (Hb cut-off <10 g/dL). Key exclusion criteria included ongoing uncontrolled bleeding, palliative status, or imminent cardiac surgery. Notably, 13% of the study population had a recent bleeding event. Meanwhile, transfusions could be delayed in patients with volume overload or those with end-stage renal disease undergoing dialysis. In the restrictive group, the composite endpoint of 30-day all-cause mortality or recurrent myocardial infarction occurred in 16.9% of patients. All-cause mortality occurred in 9.9% of patients, and cardiac death occurred in 5.5%. In contrast, the liberal strategy group experienced a lower event rate of 14.5% (relative risk (RR), 1.15; 95% confidence interval (CI), 0.99–1.34; *p* = 0.07), with 8.3% mortality (RR, 1.19; 95% CI, 0.94–1.49) and 3.2% cardiac death (RR, 1.74; 95% CI, 1.26–2.40). However, due to the lack of statistical significance in the results for the primary endpoint, decision-making should incorporate patient-specific risk, dynamic hemoglobin trends, and the risk of recurrent ischemia [25].

According to UK National Health Service (NHS) guidelines, transfusion is recommended for stable ACS patients when Hb levels fall to ≤80 g/L. For patients with active bleeding and hemodynamic instability, transfusion should be individualized and guided by serial Hb measurements once normovolemia is achieved [26]. In ACS patients presenting with significant hematuria, close hemodynamic monitoring is essential, and transfusion thresholds should be tailored to the individual. In cases of major hemorrhage where blood type is unknown, emergency transfusion with O Resus D-positive red blood cells is advised [26].

#### 3.2.2 Management of DAPT in the Context of Bleeding

Management of DAPT in the setting of acute hematuria presents a persistent clinical dilemma, as balancing the risks of thrombosis and bleeding can be challenging and potentially harmful to the patient. Patients with ACS are considered to be in a sustained prothrombotic state and are typically prescribed DAPT for 6 to 12 months. Studies evaluating DAPT with prasugrel or ticagrelor for one year have demonstrated significant reductions in ischemic events; however, these benefits come at the cost of increased bleeding risk compared to clopidogrel or aspirin monotherapy. Importantly, patients at very high risk of bleeding were excluded from these studies; as such, the findings primarily apply to patients without a very high bleeding risk [5].

The risk of bleeding associated with DAPT is estimated to be 3.4 times higher than with aspirin monotherapy [27]. Approximately 5% of patients who undergo PCI are hospitalized for bleeding complications, with most admissions occurring within the first month after treatment [28]. According to the PARIS registry, patients who were non-compliant with DAPT within 30 days post-PCI had a 2- to 3-fold increased risk of major adverse cardiac events (MACEs) or myocardial infarction [29].

Moreover, prolonged DAPT can lead to significant bleeding events, which may necessitate interruption of therapy—an action that increases the risk of recurrent MACEs [30, 31]. In one study of 1122 patients, 28.5% who discontinued DAPT within 12 months due to non-compliance or bleeding experienced a significantly higher adjusted risk of MACEs and net adverse clinical events [32]. Additionally, patients who experienced a Bleeding Academic Research Consortium (BARC) type 3 bleeding event were found to have more than twice the risk of death compared to those who experienced a myocardial infarction [33].

Bleeding episodes while on DAPT present considerable risks for morbidity and repeated hospitalizations [34]. Moreover, bleeding episodes can also lead to interruptions in DAPT, which are associated with increased thrombotic risk and adverse outcomes [35]. Thus, it is of paramount importance to involve a multidisciplinary team of specialists, including urologists, cardiologists, hematologists, and anesthetists, in the management of these patients. Given that there is a substantial anesthetic risk with increased perioperative mortality for most of these patients, the definitive surgical treatment of the bleeding cause is not always advisable in the acute setting [36]. Therefore, following a strategy to prevent further hematuria recurrences without significantly increasing the risk of thrombosis is crucial.

In the setting of mild bleeding, the continuation of DAPT is generally recommended, especially during the first month after PCI, when thrombotic risk is higher [36]. Nonetheless, clinicians may consider adjusting the treatment plan by either reducing the total duration of DAPT or substituting the current P2Y12 inhibitor with a less potent agent such as clopidogrel, depending on the bleeding and ischemic risk profile of the patient [24]. For patients experiencing moderate bleeding, temporary discontinuation of aspirin while maintaining the P2Y12 inhibitor is advised. Aspirin may be reintroduced after the bleeding has resolved and the patient has been clinically stabilized [24].

In the case of severe bleeding, the initial management step should involve de-escalation to monotherapy, preferably with a P2Y12 inhibitor. If bleeding or hematuria persists despite this adjustment, temporary cessation of all antiplatelet agents may be necessary until adequate hemostasis is achieved. Upon stabilization, DAPT may be restarted with a shorter treatment duration or a less potent antiplatelet regimen, depending on the ischemic risk of the patient [24]. This decision should be made by a multidisciplinary team that balances the risks of MACEs and bleeding.

Life-threatening bleeding mandates the immediate discontinuation of all antiplatelet therapy, regardless of the timing since PCI. Once the patient is hemodynamically stable, a careful reassessment should be performed to determine whether to resume antiplatelet treatment. Options may include restarting a single antiplatelet agent or reintroducing DAPT using a de-escalated P2Y12 strategy based on individualized risk-benefit analysis [24]. Individualized assessment, balancing the risk of MACEs and the impact of bleeding, should occur with a multidisciplinary joint team.

#### 3.2.3 Half-Life of Antiplatelets and Required Period of Stopping Antithrombotic Agents Before Intervention

3.2.3.1 Aspirin

Aspirin has a half-life in plasma of 20 minutes; however, cyclooxygenase (COX) inhibition in platelets is irreversible, so aspirin has a lasting effect equal to the life of the platelet (≈10 days). Due to the platelet turnover, platelet COX activity is restored by approximately 10% daily after a single dose of aspirin. It has been shown that normal COX activity of 20% of platelets may achieve normal hemostasis [37].

3.2.3.2 P2Y12 Inhibitors

Clopidogrel and prasugrel also bind irreversibly to the adenosine triphosphate (ADP) P2Y12 receptor on platelets with an onset of action of 2–8 hours and 0.5–4 hours, respectively. The half-life of clopidogrel is 6 hours, and the associated metabolites have a half-life of 30 minutes, with an offset of action at 5–7 days. The metabolites of prasugrel have a half-life of 7 hours and require 7–10 days to be cleared. Meanwhile, ticagrelor binds reversibly to ADP P2Y12 receptors, with an onset of action at 0.5–4 hours, a half-life of 7 hours, and metabolite half-lives of 9 hours, with offset at 3–5 days [38].

**Fig. 1.**
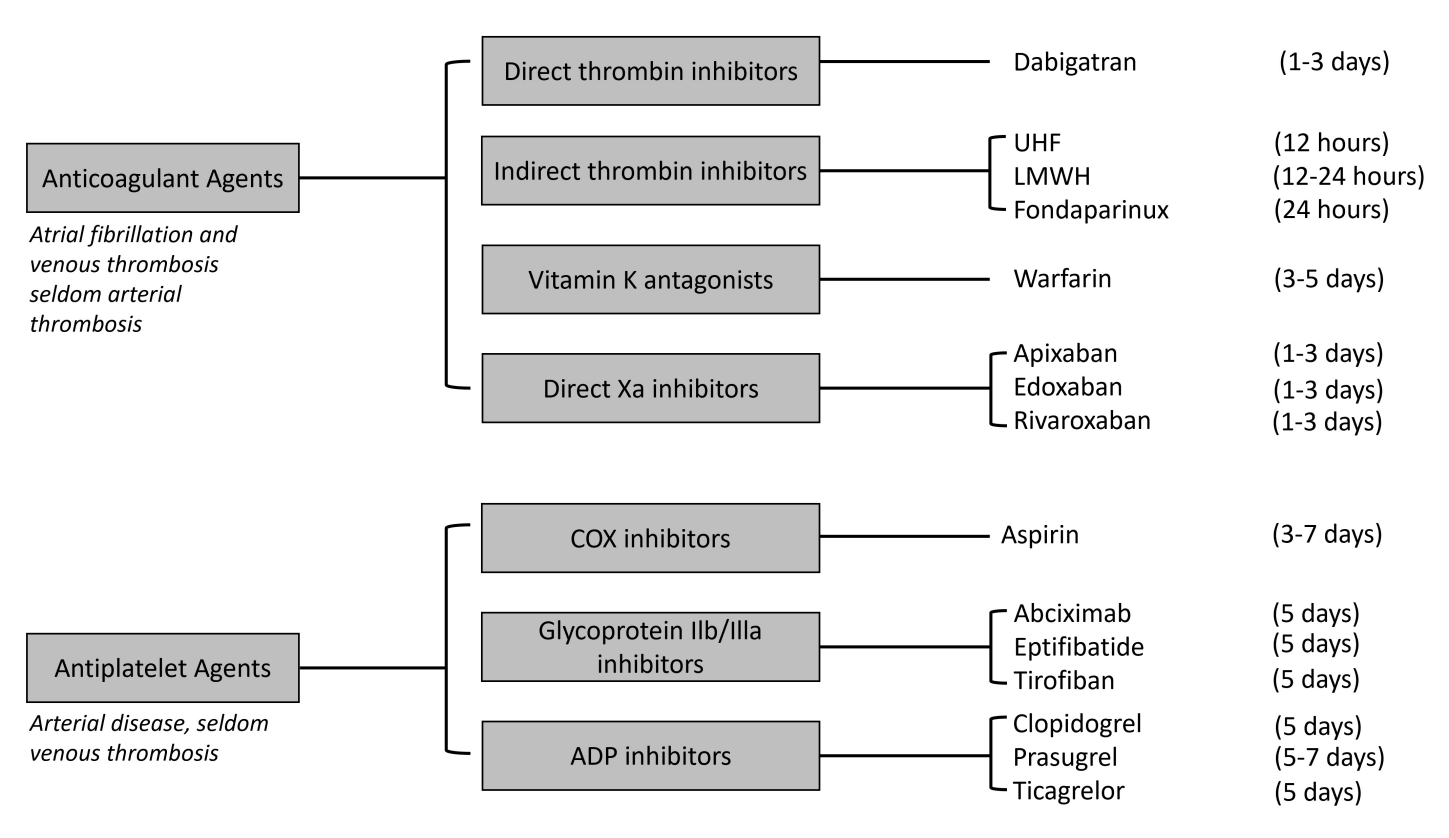
**Required period for stopping antithrombotic agents (if desired) before urological surgery—modified from European Association of Urology (EAU) guidelines: Thromboprophylaxis in Urological Surgery, 2022 [39]**. COX, cyclooxygenase adenosine triphosphate.

According to the European Urological Society guidelines, the required period of discontinuation of antiplatelet agents, if possible, before elective urological procedures is 5 days, except for aspirin (Fig. 1, Ref. [39]).

#### 3.2.4 Anticoagulant Reversal and Platelet Management in Emergency Settings

According to expert consensus for emergency departments [12], clinicians must assess the need for either coagulation factor repletion or targeted reversal therapy in cases of major anticoagulant-related bleeding. In patients receiving direct oral anticoagulants (DOACs), the decision to reverse anticoagulation must be individualized, weighing the thrombotic risk against the benefit of bleeding control. Critical to this decision is the pharmacokinetic profile of the agent—particularly the associated half-life and route of elimination. This requires knowledge of the timing of the last administered dose and any renal impairment that may delay drug clearance [12].

If drug clearance is assumed (*e*.*g*., in delayed-intake and normal renal function settings), discontinuation is often sufficient, and reversal agents are not indicated. However, if the drug was taken recently, has a prolonged half-life, or renal clearance is impaired, targeted reversal is recommended [12].

Patients with ACS are typically on DAPT, comprising aspirin and a P2Y12 inhibitor [5]. Aspirin irreversibly inhibits COX-1 and COX-2, while P2Y12 inhibitors suppress platelet aggregation by blocking adenosine diphosphate receptors [40]. Although platelet function testing and thromboelastographic (TEG) assays exist, these assays are not yet widely accessible in clinical settings [41]. Bentracimab, a monoclonal antibody targeting ticagrelor and the associated metabolite, offers rapid reversal of platelet inhibition but is not yet commercially available [42, 43]. In the REVERSE-IT phase 3 trial, Bentracimab achieved normal clotting in 94.3% of patients without major allergic reactions—100% in surgical and 83.1% in major bleeding contexts. However, limitations included a lack of a control group and underrepresentation of Black patients [44, 45] (Table 1, Ref. [27, 28, 38, 39]).

**Table 1.** **Comparative summary of oral P2Y12 inhibitors [27, 28, 38, 39]**.

Feature	Clopidogrel	Prasugrel	Ticagrelor
Binding mechanism	Irreversible; requires activation to an active metabolite by CYP enzymes, predominantly CYP2C19.	Irreversible.	Reversible.
Onset of action	2–8 hours; steady-state inhibition reached in 3–7 days.	0.5–4 hours.	0.5–4 hours.
Half-life	6 hours; its active metabolite has a half-life of approximately 30 minutes.	The active metabolite has a half-life of 7 hours.	7 hours; the active metabolite has a half-life of 9 hours.
Time to offset	Platelet aggregation and bleeding time return to baseline within 5 days after discontinuation.	7–10 days.	3–5 days; faster than both clopidogrel and prasugrel.
Implications for clinical management	Less potent than prasugrel and ticagrelor; variability in response due to CYP2C19 genetic factors.	More potent and consistent than clopidogrel, with greater inhibition of platelet aggregation.	Potent and rapid effects with faster offset, which is beneficial in patients requiring urgent procedures.
	The longer offset may increase bleeding risk in patients requiring urgent surgery.	Higher risk of bleeding, especially in patients with prior stroke or transient ischemic attack (TIA).	Significant bleeding risk, especially in patients with intracranial hemorrhage.
	The discontinuation period for elective urological procedures is 5 days.	The discontinuation period for elective urological procedures is 5–7 days.	The discontinuation period for elective urological procedures is 5 days.
	Resume within 48 hours postoperatively.		
	For spinal/epidural anesthesia or lumbar puncture, a minimum of six hours should elapse after catheter removal or regional block performance before reinitiating P2Y12 inhibitors.		
	In elective surgical cases, the multidisciplinary team should determine the appropriate timing for restarting therapy.		
	If bridging with an intravenous glycoprotein IIb/IIIa inhibitor was required preoperatively, P2Y12 inhibitors should be restarted with a loading dose.		
	Platelet transfusion: double standard dose.		Ineffective PLT transfusion due to reversible binding and redistribution.
	Efficacy can be reduced if the last intake of prasugrel was <6 h prior.		Last intake <24 h: no evidence; discuss rFVIIa.
			Last intake >24 h: platelet transfusion for partial neutralization.

CYP, cytochrome P450; CYP2C19, Cytochrome P450 Family 2 Subfamily C Polypeptide 19; P2Y12, P2Y purinoceptor 12; PLT, platelet.

Alternative interventions include desmopressin, platelet transfusion, and TXA.

3.2.4.1 Desmopressin (DDAVP)

DDAVP enhances platelet aggregation by stimulating the endothelial release of von Willebrand factor and factor VIII [42, 46]. It is recommended in neurocritical care for antiplatelet-associated intracranial hemorrhage at a dose of 0.4 µg/kg IV [47]. The efficacy of DDAVP in urological bleeding, particularly in uremic conditions, has been noted, although evidence primarily stems from small randomized or observational studies. Specifically, the administration of desmopressin has been investigated for its ability to reduce bleeding following kidney biopsy, with mixed results [48].

3.2.4.2 Platelet Transfusion

Platelet transfusion may be beneficial, particularly in aspirin-related bleeding, by replacing dysfunctional platelets. For intracranial hemorrhage, platelet transfusion is typically reserved for patients undergoing surgery, with one apheresis unit deemed sufficient [42, 47]. However, the effectiveness of a platelet transfusion depends on the pharmacokinetics of the antiplatelet agent, the timing of the last dose, and the bleeding site [49]. Platelet transfusion can counteract the effects of aspirin in a standard dose of 0.5 to 0.7 × 1011 per 10 kg of body weight. Platelet transfusion can also counteract clopidogrel and prasugrel in double dosage but is ineffective against ticagrelor due to its reversible receptor binding and redistribution [28, 50, 51, 52]. Moreover, a lag of at least 4 hours is often required post-dose to reduce the drug concentration below therapeutic thresholds, thereby limiting its emergency use.

Evidence from neurosurgical settings is conflicting: while a single-center study showed reduced hemorrhage and mortality, the PATCH multi-center trial reported increased mortality at 3 months following transfusion [49, 53]. Similar trends have been observed in gastrointestinal bleeding [54]. Potential mechanisms include thrombotic risk and proinflammatory responses to transfusion [55, 56]. Evidence for platelet transfusion in hematuria is limited and largely anecdotal.

3.2.4.3 Tranexamic Acid (TXA)

TXA may be considered in major hemorrhage in patients on antiplatelets, though its use in this context remains off-label. TXA has demonstrated benefit in reducing bleeding in trauma and surgery with a favorable safety profile [57, 58]. The recommended dosage is an intravenous administration of 1 g over 10 minutes, followed by 1 g over 8 hours. Side effects include thromboembolism and seizures [42]. In urology, TXA has shown promise in managing hematuria in polycystic kidney disease and as prophylaxis after prostate biopsy [59, 60]. A systematic review indicated no increased risk of acute renal failure with TXA use, although the number of studies was limited [61]. Bladder instillation of TXA in hematuria reduced emergency department stays, catheter duration, and readmissions [62]. Additionally, intraprostatic injection in select cases has yielded durable hemostasis [63]. The National Institute for Health and Care Excellence (NICE) guidelines support the use of TXA for critical bleeding on antiplatelet therapy when the benefit outweighs the risk, as an alternative to transfusion [35]. The European Society of Cardiology recommends the immediate use of TXA in patients with major bleeding undergoing non-cardiac surgery [36].

There is currently limited evidence regarding targeted reversal or adjunctive therapies (*e*.*g*., platelets, TXA, desmopressin) in patients with severe or life-threatening hematuria related to antiplatelet or anticoagulant use. Decisions should be multidisciplinary, involving urology, cardiology, and emergency medicine teams, and tailored to the individual clinical context.

### 3.3 Investigating the Primary Cause of Hematuria

Timely identification of the cause of hematuria is essential for appropriate treatment, particularly in patients on DAPT following ACS. As already mentioned, a diagnostic evaluation must balance the urgency of ruling out urological malignancy with the criticality of the bleeding and the thrombotic risk of stopping antiplatelet treatment.

Urological pathology is present in 44% of cases with hematuria. The differential diagnosis of macroscopic hematuria is broad, including both benign and malignant etiologies, with the latter accounting for 24% of cases [1]. Malignancies of the urinary tract, including bladder, prostate, ureter, renal pelvis, and kidney, constitute the key urological conditions. Additionally, common causes are also benign, such as urinary calculi, infections, benign prostatic hyperplasia, trauma, and iatrogenic injury usually after catheterization of urological instrumentation. Extra-urological sources include glomerular diseases of the kidneys (*e*.*g*., IgA nephropathy, lupus nephritis, different types of glomerulonephritis), coagulopathies, and drug-induced bleeding (*e*.*g*., warfarin, DOACs, NSAIDs) [1, 64].

**Fig. 2.**
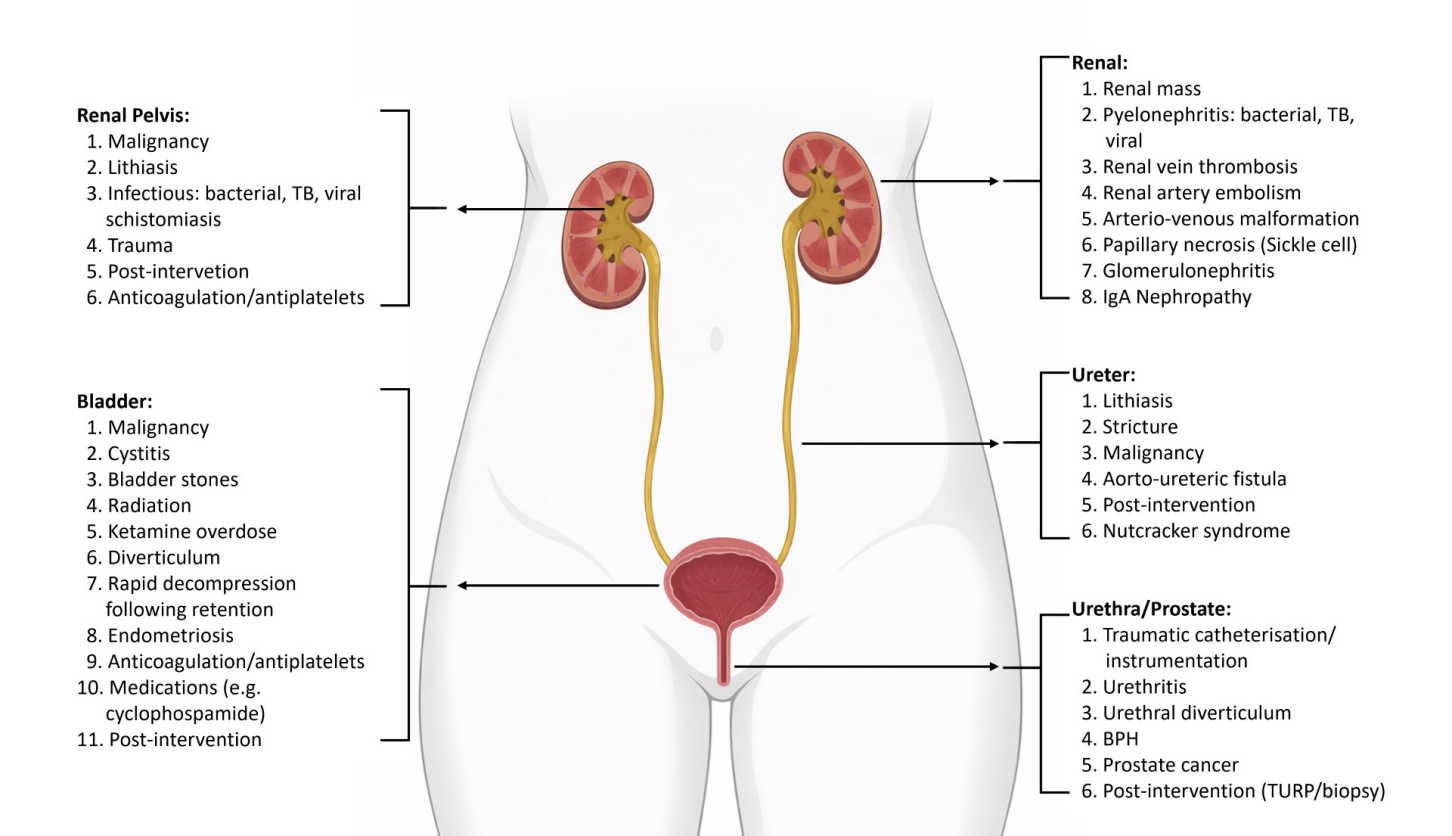
**Differential diagnosis of hematuria**. The figure was generated using BioRender (BioRender.com; Toronto, ON, Canada).

A summary of the etiological factors of visible hematuria is described in Fig. 2.

Clinically, differentiating among the different causes is challenging. Urine color, alongside a thorough urological-focused history, can significantly guide the diagnosis. As mentioned previously, bright red hematuria with clots usually suggests active bleeding in the lower urinary tract, whereas brownish or “cola-colored” urine may point toward glomerulonephritis. Further assessment and confirmation of the presence of true hematuria are currently performed via urine microscopy, in which dysmorphic red blood cells and casts suggest glomerulonephritis, whereas isomorphic RBCs imply non-glomerulonephritic urinary tract bleeding [65].

Investigations for individuals with ACS on DAPT are conducted in the same way as in the general population, based on their clinical condition and hemodynamic stability, per the American Urological Association/Society of Urodynamics, Female Pelvic Medicine & Urogenital Reconstruction (AUA/SUFU) 2025 guideline [66]. It is of imperative significance to avoid any assumptions that the antithrombotic therapy alone can be blamed for the hematuria, without assessing for other causes, such as malignancy. Interestingly, 81% of individuals in a cohort of 243 patients being treated with warfarin who developed hematuria in 2 years had an underlying urinary tract pathology [67].

Different guidelines exist for the investigation and management of visible hematuria. The European Association of Urology (EAU) [39] recommends that any episode of visible hematuria warrants a full urological evaluation, irrespective of the antithrombotic therapy. The initial workup comprises a thorough medical and urological history (including social and family history, inquiring for risk factors of both malignant and benign urological pathologies), physical examination, including per vagina and digital rectal examination in females and males, respectively, as well as urine culture. CT intravenous urography (CTU) is the gold standard for upper tract evaluation, except in patients with impaired renal function, where ultrasound and magnetic resonance urography are deemed helpful. Meanwhile, an ultrasound is also preferred in old, frail, or pregnant patients with visible hematuria. Urine cytology is suggested as it demonstrates high sensitivity in high-grade urothelial tumors, including carcinoma *in situ*. In parallel, the AUA recommends urine cytology in patients with high-risk factors (*e*.*g*., smoking, chemical exposure) [66]. In any case, urine cytology should not replace or exclude the performance of cystoscopy.

Flexible or rigid cystoscopy constitutes the gold standard for evaluating the lower urinary tract (urethra, prostatic urethra, and bladder) because it provides direct visualization of the mucosa. In the UK, the NICE guidelines [68] advocate early cystoscopy (including CTU/ultrasound) within 2 weeks under specific cancer pathways for the early investigation of hematuria. Antiplatelet treatment and antithrombotic regimens do not delay this pathway, as hematuria still carries a high diagnostic yield for urological malignancy.

#### 3.3.1 Stratification of Diagnostic Assessment Based on Hematuria Severity

The diagnostic pathway should be tailored to the severity of hematuria, hemodynamic status, and the presence of antithrombotic therapy. In cases of life-threatening hematuria, as described earlier, urgent CT abdomen/pelvis with contrast to localize the bleeding source is required. Emergency bladder irrigation and washout with wide-bore catheters is deemed the first management step before the definitive diagnosis. If bleeding is not controlled, urgent cystoscopy is recommended for both diagnostic and controlling the bleeding/treatment purposes. In cases of severe non-life-threatening hematuria, after the initial management and control of hematuria, an inpatient CTU/ultrasound alongside cystoscopy is deemed favorable for early diagnosis and subsequent treatment of the primary cause. Lastly, mild to moderate hematuria permits outpatient evaluation, assuming that the patient is hemodynamically stable. The basic work-up comprises flexible cystoscopy, CTU/ultrasound, and urine cytology based on national practice and risk factors. Notably, hemoglobin levels and coagulation parameters must be assessed and corrected promptly in all patients taking antithrombotic/antiplatelet treatment with recent ACS, as deranged hemostasis can significantly worsen bleeding from underlying urinary tract lesions and obscure the diagnostic process. Ultimately, a structured, risk-adapted approach ensures both early cancer detection and timely control of hemorrhage, thus minimizing the need to interrupt the antithrombotic therapy unnecessarily [39, 68].

3.3.1.1 Decision-Making for Targeted Therapy of the Underlying Cause

Decision-making for targeted therapy is initiated once diagnostic workup has yielded a probable cause. In select cases, such as bladder cancer, diagnosis and treatment may occur simultaneously during cystoscopy and transurethral resection of the tumor.

From a urological standpoint, therapeutic strategies for non-glomerular hematuria can be classified as invasive or non-invasive. These are tailored to the underlying pathology, which commonly includes malignancy, prostatic hyperplasia, renal/ureteric trauma, nephrolithiasis, or infection. In contrast, glomerular hematuria (often accompanied by dysmorphic red blood cells, red cell casts, and proteinuria) and uncomplicated urinary tract infections generally warrant conservative management and are more likely to be managed outside the realm of urological surgical intervention.

More invasive open interventions may include cystectomy for refractory hematuria in muscle-invasive bladder cancer and nephrectomy in life-threatening renal trauma that is beyond endovascular control. Endoscopic treatments such as cystoscopy with intraoperative clot evacuation, transurethral resection of bladder tumor (TURBT), or transurethral resection of the prostate (TURP) for prostatic bleeding aim to control bleeding and remove obstructing clots. These constitute the core initial invasive procedures [69]. Notably, urological surgeries, including endourology procedures such as prostatectomy and bladder tumor resection, and procedures with vascular organ biopsy, such as those of the kidneys or prostate, are considered surgeries with high bleeding risk.

When bleeding cannot be managed with endoscopy or surgery, patients may undergo superselective embolisation of superior or inferior vesical arteries for bladder hemorrhage, or prostatic artery embolisation for prostatic bleeding; these procedures are typically performed under general anesthesia [70, 71]. Moreover, these procedures have demonstrated high technical success with rapid cessation of bleeding and minimal major complications, even in patients unsuitable for open surgery.

In patients with refractory bleeding due to invasive cancer who are unfit for anesthesia and have not previously received radiotherapy, external beam radiotherapy (especially single-fraction or hypofractionated regimens) has proven effective for symptom palliation of gross hematuria, offering hemostasis with minimal side effects [72].

A less prominent method is chemical (fibrinolytic) thrombolysis via intravesical instillation of agents such as chymotrypsin or diluted hydrogen peroxide. These agents chemically digest intravesical clots, thereby allowing the clot to pass through the catheter or be irrigated out [73, 74]. This approach can be used when manual washouts have failed or when surgical intervention is contraindicated, but the approach requires caution due to potential urothelial irritation or gas formation.

It is important to note that most of these interventions serve a hemostatic role, aimed at temporizing bleeding rather than eradicating the primary pathology; therefore, further definitive treatment may also subsequently still be required. Additionally, prompt removal of bladder clots, either via urethral catheter or intraoperatively, is essential: retained clots promote continued bleeding through urokinase activation and subsequent local anticoagulant effects if left *in situ* [69]. Traditionally, the treatment plan is escalated from less to more invasive treatments.

In the unlikely scenario of life-threatening bleeding and simultaneous NSTE-ACS with an indication for revascularization, then the priorities for surgery should be considered individually by the expert team [36]. If anesthesia is needed, in patients with a background of ischemic heart disease, any mismatch between myocardial oxygen supply and demand can result in myocardial ischemia or even death. Several factors can exacerbate ischemic heart disease, including hypercoagulability, inflammation, hemodynamic instability, anemia, hypoxia, and withdrawal of cardiovascular medications. Additionally, unstable atherosclerotic plaques, recent coronary stent placement, and underlying cardiomyopathy can further compromise the condition of the patient.

In patients with a prior coronary stent, the physician must balance the risk of thrombosis against that of bleeding. The risk of spinal or epidural hematoma must also be considered if clopidogrel, prasugrel, or ticagrelor is continued before neuraxial anesthesia [28].

The time elapsed since stenting is a critical factor. Thrombotic risk is highest within the first 4–6 weeks following stent placement.

Interrupting or de-escalating DAPT within the first 30 days following ACS carries a very high thrombotic risk and is associated with increased mortality. Therefore, this should be avoided unless the patient faces a life-threatening hemorrhage [27, 36]. Both the ESC and ACC/AHA/ACEP/NAEMSP/SCAI guidelines strongly recommend continuing DAPT during the first 30 days, even if emergency surgery is necessary [4, 5, 75, 76].

According to the EAU guidelines, in patients at very high thrombotic risk, such as those with drug-eluting stents placed within 6 months or bare-metal stents placed within 6 weeks, postponing surgery is advised, if feasible (strong, high-quality evidence). If surgery cannot be delayed, then continuing antiplatelet therapy during the procedure is recommended. However, this carries a weaker recommendation (low-quality evidence), particularly when considering the impact of hematuria on outcomes and the practicality of surgical intervention [39].

In cases of life-threatening bleeding where the antithrombotic agents have already been stopped, and invasive treatment is required, urologists are needed to proceed with treatment urgently to control the bleeding.

In the case of severe bleeding, a multidisciplinary team should decide whether the operation can be performed under antiplatelet treatment or whether the treatment should be modified or stopped. Moreover, non-invasive alternatives, such as radiotherapy, can be explored. Meanwhile, if delaying surgery to allow the P2Y12 inhibitor to be discontinued is not possible, neuraxial (spinal/epidural) anesthesia should be avoided, and every precaution should be taken to secure hemostasis [27].

In cases of moderate or mild bleeding (according to the ESC definition), a multidisciplinary team, typically comprising a surgeon, cardiologist, and anesthesiologist, should determine whether the procedure can be postponed and performed electively or must be undertaken urgently. The necessary timeframe for managing the primary cause of hematuria should be determined to reduce thrombotic risk by increasing the interval between the ACS event and surgery. Additionally, the need to adjust the antithrombotic therapy should be evaluated. If the procedure is elective, preoperative optimization of the patient is advised.

For cases of mild to moderate hematuria, as defined in various classification systems, that resolve with conservative management, it is considered reasonable to resume standard DAPT, if previously modified, and proceed with essential hematuria investigations, including cystoscopy under local anesthetic and upper tract imaging, in the absence of other high bleeding risk factors [3].

**Fig. 3.**
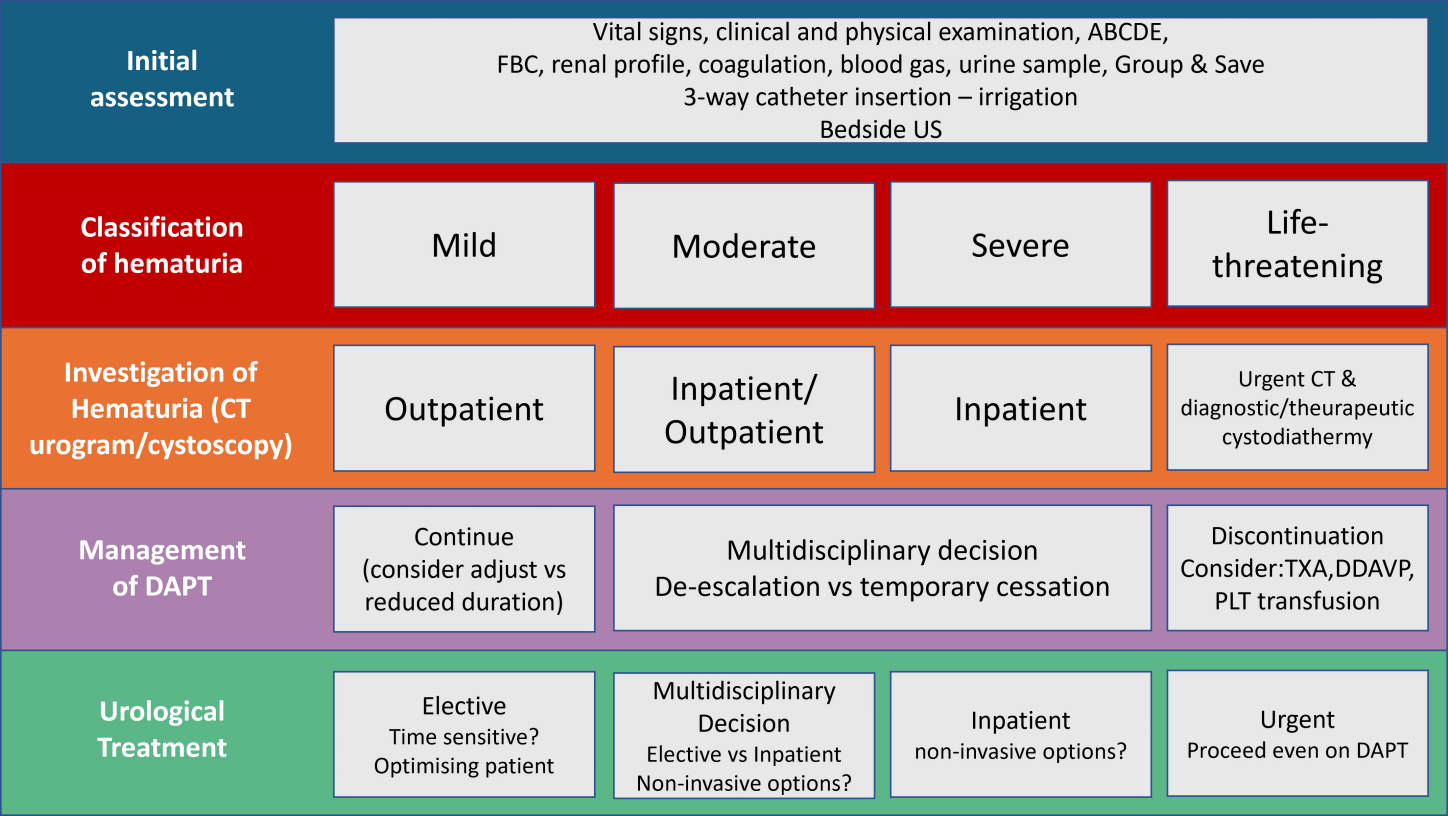
**Suggested diagnostic and therapeutic algorithm in patients with hematuria following ACS**. ACS, acute coronary syndrome; US, Ultrasound; FBC, Full blood count; DAPT, Dual Antiplatelet treatment; CT, Computed Tomography scan; TXA, tranexamic acid; DDAVP, desmopressin.

**Fig. 4.**
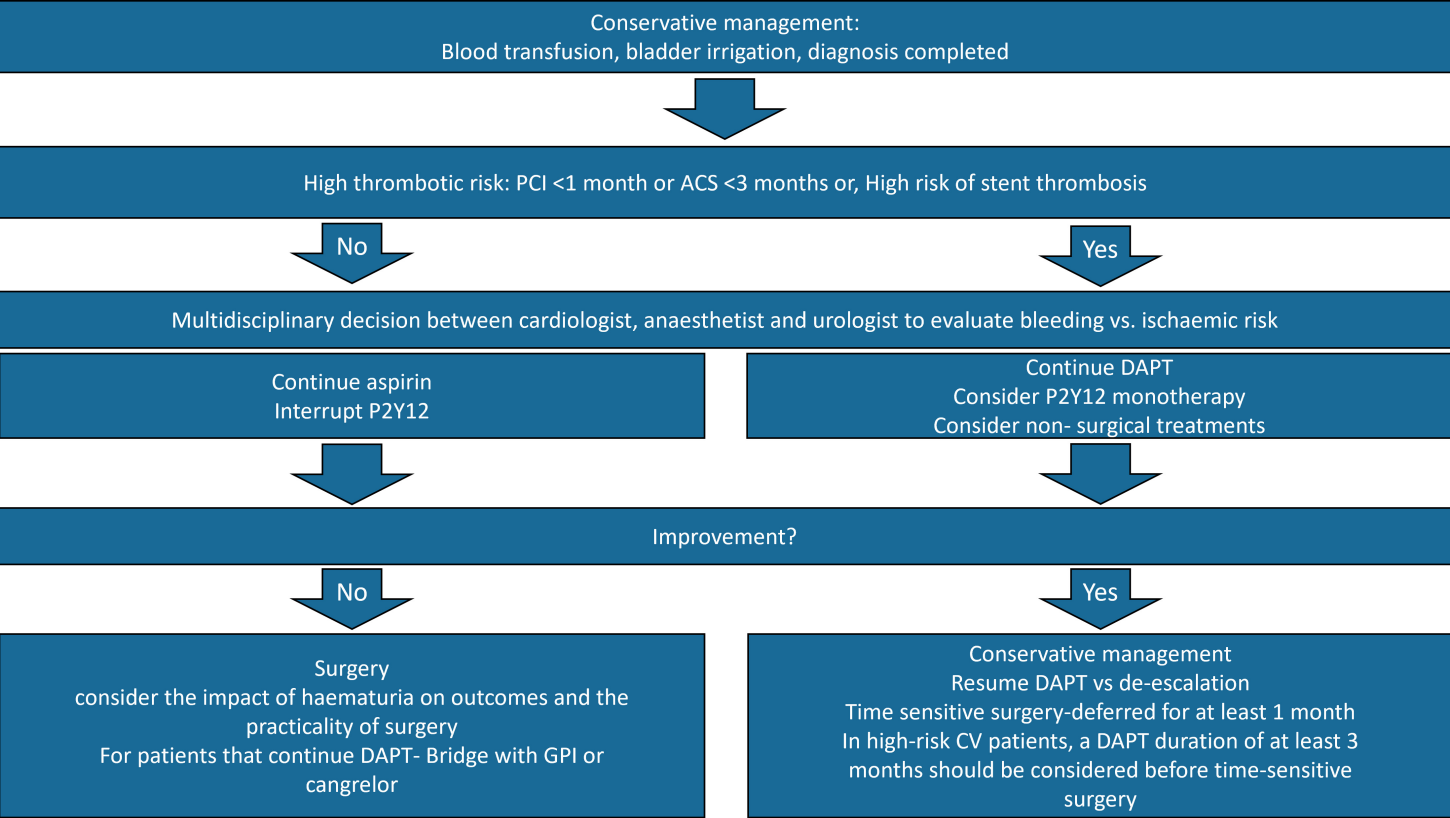
**Multidisciplinary approach in moderate/severe hematuria involving cardiology, urology, and anesthesiology teams**. Management must be individualized, considering patient-specific factors, dynamic clinical evolution, and the balance between cardiac and urological priorities. It is also important to note that no dedicated guidelines or primary studies currently exist regarding the management of urological procedures or active hematuria in ACS patients. Consequently, many recommendations are extrapolated from general guidelines [36, 39, 77]. Due to the scarcity of data and the lack of specific studies for hematuria, the strength of recommendations cannot be formally graded. PCI, Percutaneous Coronary Intervention; P2Y12, P2Y purinoceptor 12 (P2Y12) inhibitor; GPI, Glycoprotein IIb/IIIa inhibitors.

For elective surgeries following elective PCI, it is recommended to defer surgery for at least 6 months, and for 12 months after ACS. After elective PCI, time-sensitive non-cardiac surgery (NCS) should ideally be delayed until at least 1 month of DAPT has been completed. For patients with ACS or high ischemic risk features, a minimum of 3 months is recommended (Fig. 3 and Fig. 4, Ref. [36, 39, 77]).

High ischemic risk features include a history of recurrent myocardial infarction, a history of stent thrombosis despite antiplatelet therapy, left ventricular ejection fraction <40%, poorly controlled diabetes, severely impaired renal function or hemodialysis, recent complex PCI, stent malposition, or residual dissection.

In patients with prior PCI, aspirin may generally be continued, unless a surgery with a high bleeding risk is planned. In such cases, aspirin should be stopped 7 days before surgery. If PCI was not performed, aspirin should be discontinued 3 days before surgery to help reduce bleeding risk. If surgery can be safely postponed, patients with ACS should undergo diagnostic and therapeutic interventions in accordance with standard ACS management protocols [36].

In situations where the multidisciplinary team determines that both aspirin and the P2Y12 inhibitor must be stopped, bridging therapy with intravenous glycoprotein IIb/IIIa inhibitors may be considered [27]. In selected patients at high thrombotic risk who require temporary discontinuation of oral P2Y12 inhibition for urgent surgical procedures, short-acting intravenous P2Y12 receptor blockade, such as cangrelor, may be considered as a bridging strategy. The rapid onset and offset of platelet inhibition of this blockade allow maintenance of antithrombotic protection while minimizing perioperative bleeding risk, particularly in procedures where early hemostasis is achievable. Bridging decisions should be individualized and undertaken in close collaboration with cardiology, surgical, and anesthesiology teams, balancing ischemic risk, procedural bleeding risk, and anesthetic approach [78, 79, 80].

In conclusion, patients with a recent history of ACS face increased risks when undergoing general anesthesia. Meanwhile, careful planning, risk assessment, and surgical timing are vital to ensure patient safety and improve surgical outcomes.

3.3.1.2 Resumption of Antiplatelet Medications Following Hematuria Episodes

Decisions regarding DAPT management following a hematuria episode largely depend on the timing of the hematuria in relation to the ACS, the severity and underlying cause of hematuria (and whether it has been definitively addressed), the risk of future significant bleeding requiring invasive treatment, patient comorbidities, and individual preferences.

The Academic Research Consortium for High Bleeding Risk (ARC-HBR) has defined criteria to identify patients undergoing PCI at high bleeding risk, including major and minor criteria such as advanced age, prior bleeding episodes, liver or kidney disease, and anemia [81]. Additionally, several validated bleeding risk scores have been developed to quantify bleeding risk and potentially guide decisions about antiplatelet therapy [81, 82].

A Korean national cohort study involving 325,417 patients undergoing PCI found that patients with a high bleeding risk had a 3.12-fold higher risk of major bleeding compared to those who did not (95% CI: 3.04–3.21). Furthermore, patients with a high bleeding risk had a 2.5-fold increased risk of cardiac death, myocardial infarction, or ischemic stroke, with the majority of adverse outcomes attributable to cardiac death (27.7% vs. 9%; hazard ratio (HR): 3.73; 95% CI: 3.66–3.79) [83, 84].

Patients experiencing Bleeding Academic Research Consortium (BARC) type 3 bleeding events have been shown to have more than double the risk of death (HR: 2.71; 95% CI: 2.64–2.77) compared to those with myocardial infarction (HR: 1.33; 95% CI: 1.28–1.39). These findings remained consistent in subgroup analyses of patients with a high bleeding risk. The study authors suggested that bleeding risk should take precedence over ischemic risk in patients with a high bleeding risk [33].

A pooled analysis of eight randomized controlled trials, including 14,963 patients, demonstrated that those with a high bleeding risk (defined by a PRECISE-DAPT score ≥25) did not derive significant mortality or ischemic benefit from prolonged DAPT, regardless of ACS or PCI complexity. On the contrary, these patients experienced more bleeding events with prolonged therapy. These findings suggest that DAPT duration should be individualized based on ischemic risk and clinical presentation in patients with a high bleeding risk [85].

In general, following the discontinuation of antiplatelet therapy before surgery, restarting therapy as soon as possible (ideally within 48 h) is recommended. Aspirin is typically continued perioperatively; however, if discontinued, aspirin administration should be restarted as soon as clinically feasible [36]. P2Y12 inhibitors should be resumed within 48 hours postoperatively. For patients undergoing neuraxial (spinal/epidural) anesthesia or lumbar puncture, a minimum of six hours should elapse after catheter removal or regional block performance before reinitiating P2Y12 inhibitors. In elective surgical cases, the multidisciplinary team should determine the appropriate timing for restarting therapy. If bridging with an intravenous glycoprotein IIb/IIIa inhibitor was required preoperatively, P2Y12 inhibitors should be restarted with a loading dose [27].

Continuation of DAPT is crucial during the first 30 days post-ACS due to high thrombotic risk and should be maintained [75, 76]. However, a more personalized approach may be adopted beyond this period. In cases of mild to moderate hematuria that resolve with conservative management, resuming standard DAPT may be appropriate, along with essential hematuria investigations, such as cystoscopy, under local anesthetic and imaging of the upper urinary tract, provided no other high bleeding risk factors exist [3]. For patients with recurrent hematuria requiring multiple hospitalizations, severe bleeding necessitating transfusion or invasive intervention, or multiple ARC-HBR-defined risk factors, deviation from standard DAPT to more conservative regimens should be considered in consultation with cardiology specialists [75, 76, 81]. This is particularly important if the underlying cause of bleeding has not been adequately addressed due to concurrent high anesthetic risk.

Several trials have evaluated the safety of shortened DAPT followed by potent P2Y12 inhibitor monotherapy. These studies have consistently demonstrated a reduction in bleeding events without a significant increase in thrombotic complications or mortality [86, 87]. Current ESC guidelines provide a class IIa recommendation to switch to ticagrelor monotherapy after 3–6 months of DAPT in patients at low thrombotic risk and a class IIb recommendation to switch after just 1 month in those at high bleeding risk [4]. Similarly, the ACC/AHA/ACEP/NAEMSP/SCAI guidelines recommend switching to ticagrelor monotherapy after 1–3 months of DAPT in high-risk patients with bleeding (class I) [5].

Although positive results have also been observed in studies evaluating clopidogrel monotherapy following shortened DAPT, concerns remain regarding variability in platelet inhibition among patients [88, 89]. Another alternative is DAPT de-escalation, where a potent P2Y12 inhibitor is substituted with clopidogrel in patients with a high bleeding risk. This approach can be guided by platelet function testing to ensure adequate antiplatelet response [90]. However, the evidence supporting de-escalation is less robust, and current ESC and ACC/AHA/ACEP/NAEMSP/SCAI guidelines provide weaker recommendations for this strategy [5, 36].

The association between antiplatelet agents and hematuria was evaluated in a review of 45,525 patients, which found that antiplatelet agents were 76 times less likely to cause hematuria than anticoagulants. Notably, combining two antiplatelet agents did not increase the risk of hematuria (0.13%). Meanwhile, hematuria was more common with prophylactic unfractionated heparin (UFH) and low-molecular-weight heparin (LMWH) than with antiplatelet agents, but remained lower than with anticoagulants. Directional differences are suggested across studies in the reported effects of aspirin versus anticoagulants on hematuria, although limited comparability between study designs and populations necessitates cautious extrapolation. The hematuria risk for dual therapy with aspirin plus ticagrelor or aspirin plus clopidogrel remained at 0.13% (19/14,056). Among patients with visible hematuria, clopidogrel was more frequently associated with major hematuria compared to aspirin (33.3% vs. 28.3%; odds ratio (OR): 1.2; 95% CI: 0.35–4.4), whereas ticagrelor was associated with a lower risk (17.3%; 95% CI: 0.16–1.69) [42]. Population demographics, comorbidities, and medication adherence may influence these differences. The precise molecular interactions between antiplatelet agents and the urothelium remain unclear.

Potential drug–drug interactions (DDIs) involving P2Y12 inhibitors and other medications have been identified. For example, pantoprazole and P2Y12 inhibitors share a CYP450-mediated activation pathway, although the clinical relevance of this interaction remains uncertain [1]. Similarly, clopidogrel and simvastatin, both metabolized by CYP3A4, may interact, although without evident clinical impact [87]. No significant pharmacokinetic interactions have been reported between phenprocoumon and statins [91, 92]. A study examining DDIs in patients with gross hematuria found no significant association between DDIs and duration or volume of fluid irrigation, suggesting DDIs were not predictive of clinical outcomes [93].

Despite several classifications of bleeding [18, 19, 20], these are non-site-specific, making comparisons of prognosis, incidence, and management plans across studies difficult. Furthermore, the current classification of hematuria is based on clinical findings (color) and does not include laboratory results. A scoring system that incorporates both clinical signs and laboratory findings in patients with different causes of hematuria and predicts 30-day mortality is required. Re-evaluation of current hematuria definitions and the creation of a scoring system will facilitate further studies and enable comparisons across various urological causes of hematuria, anticoagulation treatments, and management strategies.

Moreover, there is a lack of data regarding reversal and antiplatelet management in patients with hematuria. Most studies on desmopressin originate from small cohorts of patients undergoing kidney biopsy [48]. Similarly, information regarding platelet transfusion is derived from studies on other bleeding sites, such as intracranial [49, 53] or gastrointestinal [54]. In contrast, the use of TXA in hematuria patients has been investigated in only a few studies of elective procedures, such as prostate biopsy [59, 60]. Furthermore, randomized studies investigating different DAPT modification strategies in patients with hematuria, with stent thrombosis or cancer diagnosis as endpoints, are also required. These studies should further evaluate outcomes in a time-sensitive manner and differentiate between conservative management of hematuria and invasive treatment of the primary cause. In conclusion, less rigid adherence to standard DAPT protocols may be warranted in patients with a high bleeding risk to reduce hemorrhagic complications and related morbidity. However, such strategies should be viewed as compromises appropriate only for selected high-risk patients and should not be applied indiscriminately to the broader post-ACS population.

## 4. Conclusion

Managing hematuria in patients receiving antithrombotic therapy after ACS is a complex and high-stakes clinical scenario. The dual imperatives of controlling hemorrhage and maintaining adequate antithrombotic protection demand early multidisciplinary collaboration between urology, cardiology, hematology, and anesthesia teams.

A systematic approach, beginning with hemodynamic stabilization, urgent evaluation for underlying pathology, and careful stratification of bleeding severity, can guide safe and effective decision-making. Whenever possible, DAPT should be maintained during the critical first month post-PCI, with de-escalation or temporary discontinuation reserved for severe or life-threatening bleeding. Definitive treatment of the hematuria source should be performed promptly when feasible; meanwhile, patients at high risk of bleeding may benefit from shorter DAPT courses or monotherapy, as supported by current guidelines. Ultimately, patient-centered, evidence-informed, and risk-adapted care remains key to improving both urological and cardiovascular outcomes in this vulnerable population.
